# The Physiology of Lived Experience: And then there was one – the story of a radical nephrectomy

**DOI:** 10.1113/EP093520

**Published:** 2025-12-07

**Authors:** Michael J. Shattock

**Affiliations:** ^1^ School of Cardiovascular and Metabolic Medicine and Sciences, BHF Centre of Research Excellence King's College London London UK

It is not every day that a PhD thesis saves your life. It was the last week of June 2024 and for the last few days I had been experiencing what felt like an intercostal muscle strain which, of course, I ignored. It was a normal Friday afternoon and I was sitting reading at my desk in the hospital when this pain morphed into an acute shortage of breath. This was not the fault of the thesis, which was beautifully written and a credit to an excellent student. However, after about 15 min, this breathlessness got progressively worse. As a cellular physiologist working in a hospital, there is always a clinical colleague at hand – except, of course, when you need one. So, with the aid of a non‐clinical colleague (an expert in murine molecular imaging!), I struggled downstairs to A&E and threw myself into the care of our wonderful NHS.

They say a little knowledge is a dangerous thing and on my way to A&E I mentally reviewed the physiology of the control of breathing, the causes of dyspnoea and the best‐ and worst‐case scenarios. I recalled ventilation is controlled by the medullary respiratory centres and is modulated by mechano‐receptors in the chest wall and central and peripheral chemoreceptors (Hall, 2015). I am not intrinsically a worrier so I had already ruled out an acute myocardial infarction, heart failure or airway obstruction, but could not help reviewing in my mind the possibility of a pulmonary embolism or a pneumothorax (Parshall et al., 2012). However, the triage staff at A&E were taking no chances and an ECG and troponin blood test quickly ruled out cardiac involvement (ESC Scientific Document, Group), a normal d‐dimer ruled out a pulmonary embolism (Thomas et al., 2024) and a chest X‐ray showed no signs of a pneumothorax (Iqbal et al., 2025). Nonetheless I remained breathless and, although it did not feel like it at the time, in retrospect this was the first of many lucky accidents.

In June 2024 junior doctors in the UK were on strike over pay and conditions. Ironically, this was my second stroke of luck. In the absence of junior doctors, I was seen by a senior consultant who was not happy with the lack of a diagnosis and asked for a CT scan – her view being that lesions that were unclear on X‐ray may be more visible on a CT (Webb & Higgins, 2015). The overworked radiographer was not keen but was outranked and over‐ruled, and a CT was duly performed. Sometime later (it was, after all, a Friday evening in a very busy A&E in central London), the CT results came back with an all clear on the thorax – no signs of anything to worry about. However, the CT did reveal a large 6 cm mass on my left kidney – just visible in the field of view (more luck?) – and calcification of at least two coronary arteries. Oh dear.

I was immediately referred to the cardiologists for an exercise test (on Monday morning) and the urologists to discuss all things renal (on Tuesday morning). As the dyspnoea had abated, I was discharged. At home over the weekend, I was left to come to terms with a cancer diagnosis and, perhaps less pressingly, to ponder what causes acute dyspnoea? With regard to the latter the most plausible explanation, despite declaring above that I'm not a panicker, was that I had indeed pulled a muscle, hyperventilated and had an acute panic attack.

## 1 THE PHYSIOLOGY OF PANIC ATTACKS AND DYSPNOEA

Classically, a panic attack represents an acute episode of fear, real or imagined, accompanied by a surge of autonomic activity – mediated through the amygdala, hypothalamus and brainstem arousal systems (Gorman et al., 2000). Activation of the sympathetic nervous system produces tachycardia, sweating, tremor and a powerful sense of impending doom (Pohl et al., 1988). I was certainly anxious and possibly hyperventilating despite my perception of breathlessness. The ventilatory response to ‘panic’ depends on the stimulus invoking it (Tipton et al., 2017) and, in my case, I am not entirely sure what that was! However, dyspnoea is not merely a mechanical event but also involves perception – where mismatched sensory input from the effort of breathing does not correspond to the afferent feedback from the lungs and chest wall (Parshall et al., 2012). So, when motor command and sensory feedback diverge, for example when ventilation exceeds metabolic demand or when airflow is restricted, the sensation of breathlessness is amplified (Parshall et al., 2012).

In my case, it is perfectly reasonable to suppose that this increase in respiration led to hypocapnia and alkalosis and this increase in plasma pH can subsequently reduce respiratory drive (Hall, 2015). Hypocapnia also causes cerebral arterial vasoconstriction and an associated decrease in blood flow resulting in light‐headedness, dizziness and anxiety (Hall, 2015). In my case, was that all it was? Well, Ockham's Razor says it probably was – and it is, after all, the most benign of diagnoses. What I really should have done is saved the NHS time and money and breathed in and out of a paper bag and resumed reading the thesis! However, that would have left me completely unaware of the incidental findings of coronary arterial disease and a renal carcinoma – conditions cumulatively responsible for approximately 71,000 deaths annually in the UK (British Heart Foundation, 2025; Cancer Research UK, 2025).

## 2 THE PHYSIOLOGY OF CALCIFIED BUT STABLE CORONARY ARTERY PLAQUES

**FIGURE 1:**
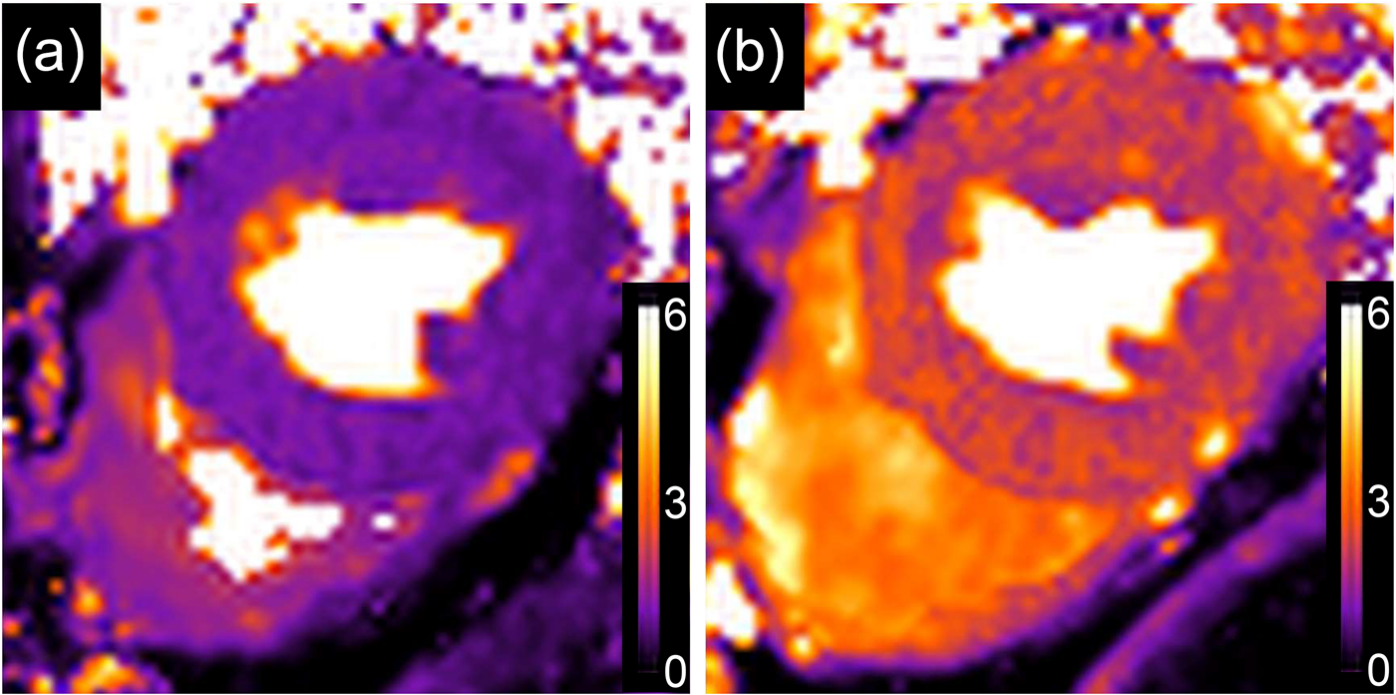
Quantitative myocardial perfusion mapping (mL min^−1^ g^−1^ of myocardium). (a) Rest perfusion map. (b) Adenosine stress perfusion map. The subject is the author.

The exercise testing for which I was destined is a simple non‐invasive method to test for coronary flow reserve in patients with coronary artery lesions. Readouts from such tests include measurable changes in the ST‐segment of the ECG, chest pain or angina, haemodynamic (blood pressure) changes and/or arrhythmias (Gibbons et al., 1997; Mark et al., 1991). So, on the Monday morning, I was duly wired to a 12‐lead ECG and run through an incremental ‘Bruce’ protocol. Embarrassingly I failed to complete the exercise protocol – perhaps due to persistent breathlessness but possibly also due to old age and a lack of physical fitness – no excuses. So, I was referred for an MRI Perfusion Stress Test, which was performed on the Wednesday morning. During this test intravenous access allows a gadolinium contrast agent to be injected and baseline measurements made of coronary artery and myocardial perfusion (Hamilton‐Craig et al., 2023). An infusion of adenosine is used to induce vasodilation and tachycardia, and coronary perfusion reassessed – with areas of delayed or reduced contrast uptake indicating ischaemic regions. Thankfully, my coronaries were patent with normal perfusion and normal flow reserve (see Figure 1).

Coronary artery plaques are indicative of atherosclerosis, a chronic inflammatory process triggered by endothelial injury, lipid accumulation and immune cell infiltration (Kong et al., 2022). Plaques typically form as a subendothelial lesion containing foam cells (lipid laden macrophages), hyperplastic smooth muscle cells and extracellular matrix. Over time, plaques may undergo calcification, following osteogenic signalling in vascular smooth muscle cells (involving BMP‐2, Runx2, etc.) (Durham et al., 2018). Calcified plaques are generally considered stable because the calcification often coexists with a thick fibrous cap, minimal lipid core and low inflammatory activity (Onnis et al., 2024). Plaque rupture is, however, dangerous – an event responsible for acute myocardial infarction or unstable angina; however, the role of calcification in stabilising plaques is complex. While homogenous ‘sheet’ calcification stabilises the plaque, punctate or ‘spotty’ calcification can increase plaque vulnerability (Jinnouchi et al., 2020; Onnis et al., 2024).

In my case, a history of taking a 3‐hydroxy‐3‐methylglutaryl‐coenzyme A reductase inhibitor (atorvastatin) almost certainly stabilised these calcified plaques. Statins lower low density lipoprotein cholesterol, the primary driver of atherosclerosis, and enhance plaque stability thanks to wide ranging pleiotropic effects including reducing pro‐inflammatory cytokines, reducing macrophage activity, improved endothelial function, promoting fibrous cap thickening, and decreasing platelet activation and coagulation factors (Liao & Laufs, 2005). So, cardiac complications dealt with, I was shunted on to the urologists.

## 3 THE PHYSIOLOGY OF A RADICAL NEPHRECTOMY

At the time of the incidental finding I was, in terms of renal function, normal and asymptomatic. No protein or blood in my urine, no unexplained back pain and a glomerular filtration rate (GFR) of ∼86 mL min^−1^. While GFR varies between individuals, a filtration rate of 86 mL min^−1^ represents a perfectly respectable kidney function for someone of my age and body size (Wetzels et al., 2007). I was told at the time that I could have remained asymptomatic for years before presenting with fatigue, haematuria and widespread lethal tumours. In fact, the first tangible symptoms may have been lung metastases and coughing‐up blood. Not great. In my case, this early and incidental finding may have literally been a lifesaver.

**FIGURE 2:**
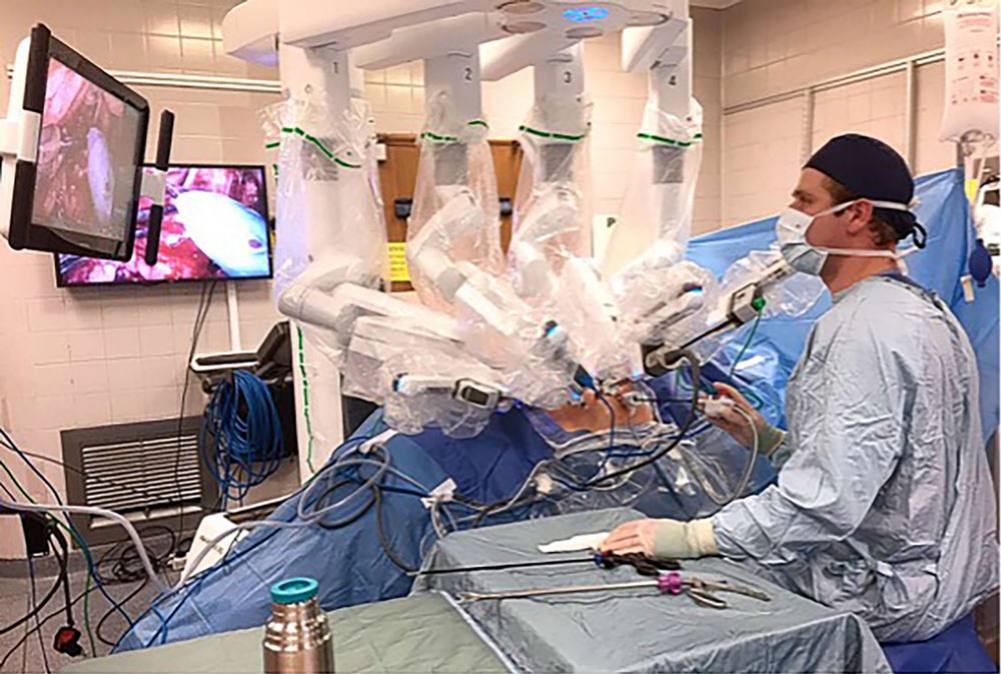
The *da Vinci* robot and surgical team at Guy's and St Thomas's NHS Foundation Trust.

The size and location of the primary tumour suggested the best option was a radical nephrectomy. The tough fibrous cap encapsulating the kidney acts to delay the spread of cancer cells and, while the blood supply remains uncompromised in this solid tumour, when caught early, the spread of tumour cells can be contained. On 24 July 2024 a robot called *da Vinci* (Figure 2) performed a radical nephrectomy in the skilled hands of my amazing urology surgeon and her team.

Here is some more interesting physiology. Even though theoretically my GFR should have halved, 2 days after the surgery my estimated GFR was 52 mL min^−1^. One month later, my GFR had risen further to 55 mL min^−1^. That got me interested as a physiologist. How does your remaining kidney ‘know’ and how does it step up its game?

**FIGURE 3:**
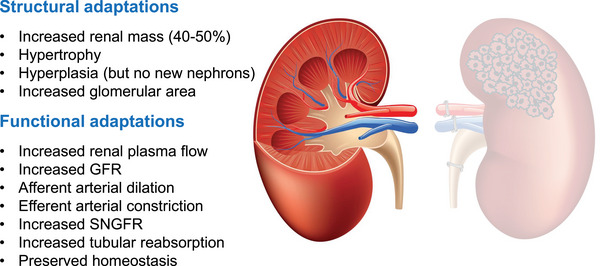
Remodelling and physiological adaptations of the remaining kidney following a unilateral total nephrectomy.

Within days to weeks, the remaining kidney undergoes compensatory hypertrophy and hyperfiltration, increasing its individual nephron GFR to increase total renal function. This adaptation occurs through both structural and functional mechanisms (Figure 3). Structurally, the mass of the remaining kidney increases by up to 40–50% within 1–2 weeks (Cleper, 2012; Hayslett, 1979; Rojas‐Canales et al., 2019). Histologically, this reflects hypertrophy of existing nephrons (particularly proximal tubules and glomeruli) and expansion of the glomerular capillary surface area. This hypertrophic process is at least in part stimulated by local release of insulin‐like growth factor 1 (IGF‐1) and transforming growth factor‐β (TGF‐β) (Mulroney et al., 1992). Interestingly, no new nephrons are formed (nephrogenesis ceases soon after birth) (Cleper, 2012) and so this adaptation depends on the existing nephrons increasing in size and capacity. Functionally, haemodynamic changes also increase the filtering capacity of a single nephron (SNGFR). Afferent arteriolar dilation increases blood flow into glomeruli while efferent arteriolar constriction (mediated by local angiotensin II release) increases glomerular capillary hydrostatic pressure (Chapman et al., 2010). Together, these raise the net filtration pressure and thus SNGFR. In total, the renal plasma flow to the remaining kidney increases by 40–60% mediated by these changes in haemodynamics and an overall reduction in renal vascular resistance driven by local nitric oxide, prostaglandins and diminished sympathetic tone (Rojas‐Canales et al., 2019). Following a nephrectomy, hyperfiltration in the remaining healthy kidney is, at least in the short to medium term, largely stable and adaptive (Provoost & Brenner, 1993). However, this is not so in chronic kidney disease or diabetes where hyperfiltration can lead to progressive scarring and nephron loss. In chronic kidney disease, this vicious spiral of hyperfiltration promotes further injury and is thought to be a key feature of disease progression (Brenner et al., 1996).

Following nephrectomy, adaptive hyperfiltration in the healthy remaining kidney means that tubular reabsorption rates must also increase proportionally in order to maintain sodium, water and solute balance (Layton et al., 2017; Shirley & Walter, 1991). The proximal tubule hypertrophies, enhancing its resorptive capacity (Layton et al., 2017; Shirley & Walter, 1991). The increased flow through the nephron would otherwise lead to greater sodium excretion, but adaptive mechanisms, including tubuloglomerular feedback, reset to preserve homeostasis. This remarkable adaptation allows me to function perfectly well on my one remaining kidney.

## 4 AND FINALLY

The last year has been a roller‐coaster. It may be unusual to hear a cancer patient say ‘I have been incredibly lucky’, but I have been incredibly lucky! I have a long list of things for which I am grateful: a junior doctors’ strike, a persistent A&E consultant, my kidney making a guest appearance in a thoracic CT scan, an observant radiologist, an attentive and skilled cardiologist, access to world‐class MRI imaging, a brilliant surgeon and a steady‐‘handed’ robot called *da Vinci*, and the wonderful care provided by the UK National Health Service – ‘free at the point of delivery’. But mostly, I would not be here without the amazing resilience and adaptive physiology of the human body – it never ceases to inspire me. Finally, I need to thank my PhD student as without her literally breathtaking thesis, my prognosis could have been so much worse.

## AUTHOR CONTRIBUTIONS

Sole author.

## CONFLICT OF INTEREST

None declared.

## FUNDING INFORMATION

None.
